# Pulmonary arterial size and response to sildenafil in chronic thromboembolic pulmonary hypertension

**DOI:** 10.1016/j.healun.2009.12.014

**Published:** 2010-06

**Authors:** Mark R. Toshner, Deepa Gopalan, Jay Suntharalingam, Carmen Treacy, Elaine Soon, Karen K. Sheares, Nicholas W. Morrell, Nicholas Screaton, Joanna Pepke-Zaba

**Affiliations:** a Department of Medicine, University of Cambridge, Addenbrooke's Hospital, Cambridge, UK; b Department of Respiratory, Pulmonary Vascular Diseases Unit, Papworth Hospital NHS Trust, Papworth Everard, Cambridgeshire, UK; c Royal United Hospital, Bath, UK

**Keywords:** pulmonary hypertension, chronic thromboembolic pulmonary hypertension

## Abstract

**Background:**

Relative area change (RAC) of the proximal pulmonary artery is a measurement of pulmonary artery distensibility and has been shown to correlate with vasoreactivity studies in patients with idiopathic pulmonary arterial hypertension. We have previously noted a relationship between invasive hemodynamic vasoreactivity testing and long-term response to sildenafil in patients with inoperable chronic thromboembolic pulmonary hypertension (CTEPH). We therefore set out to determine whether RAC can provide useful correlatory non-invasive data.

**Methods:**

Patients recruited to a randomized, controlled trial (RCT) of sildenafil at 40 mg 3 times daily underwent additional magnetic resonance imaging (MRI) at the baseline of the trial. Eighteen patients had an MRI that led to a diagnosis of inoperable distal CTEPH or significant residual CTEPH post-operatively. The primary end-point was improvement in 6-minute walk test (6MWT) with secondary end-points of right heart catheterization–based hemodynamics, N-terminal pro–brain natriuretic peptide (NT pro-BNP) and functional class. RAC assessed by MRI was correlated with trial end-points.

**Results:**

Fourteen subjects with baseline MRI completed the protocol. RAC was the only baseline variable that correlated at 1 year to the primary end-point of improvement in 6MWT (*r* = 0.7, *p* = 0.006), and also to a change in NT pro-BNP (*r* = 0.59, *p* = 0.03). Using a cut-off of RAC over 20% there was an 87.5% sensitivity (95% confidence interval [CI]: 45% to 100%) and a 66.7% specificity (95% CI: 22% to 96%) for an improvement in 6MWT of >40 meters.

**Conclusions:**

RAC correlates with functional response to sildenafil, as measured by the 6MWT, and improved heart function, as measured by NT pro-BNP. RAC shows potential in understanding and possibly predicting treatment response.

Chronic thromboembolic pulmonary hypertension (CTEPH) is considered to represent a rare consequence of acute pulmonary embolic disease.^1^ After an acute event, thrombus fails to adequately resolve and organizes, resulting in chronic obstruction of the pulmonary vasculature. If untreated, this causes progressively worsening pulmonary hypertension, right ventricular dysfunction and death.^2^ Patients are routinely treated with anti-coagulation and supportive therapy, but until recently the only proven treatment option effectively targeting pulmonary hypertension has been pulmonary endarterectomy (PEA). If a patient has surgically inaccessible disease or significant post-operative disease, options are limited. Recently, we reported the results of a randomized, controlled trial (RCT) showing that targeting vascular remodeling in CTEPH with sildenafil may be beneficial.^3^ Evidence is also accumulating from a multicenter RCT of bosentan^4^ and open-label trials of sildenafil, prostanoids and bosentan.^5–9^ As these trials have not shown a large, clear-cut response in all subjects, it is apparent that we need better tools to understand who responds and why.

Within our cohort of patients involved in the RCT of sildenafil there was a significant degree of vasoreactivity on formal vasodilator challenge studies with inhaled nitric oxide and intravenous sildenafil at baseline,^10^ which modestly correlated with improvement in pulmonary vascular resistance (PVR) at 1 year.^3^ Previously it was hypothesized that this vasoreactivity reflects similarities to idiopathic pulmonary arterial hypertension (PAH) in distal pulmonary artery remodeling and histopathologic characteristics in small vessels.^11^ This focus on the similarities of the distal vasculature overlooks the importance of the proximal vasculature, particularly its response to downstream remodeling and coupling to the right ventricle. In normal physiology the majority of vasoreactivity is in the resistance vessels in the distal vascular bed; however, it is well established that, under conditions of vascular dysfunction, there are also alterations in the vasoactive ability of proximal arteries.^12,13^

Relative area change (RAC) of the proximal pulmonary artery is a measurement of pulmonary artery distensibility and has been shown to correlate well with vasoreactivity studies in patients with idiopathic pulmonary arterial hypertension (IPAH).^14^ It measures the change in relative area between systole and diastole in the pulmonary arteries using MRI or echocardiography.^14–16^ PA distensibility is of clear importance in disease progression with a higher distensibility index, and therefore preserved compliance predicting survival in a cohort of patients with IPAH and associated causes of PAH.^16^ We therefore hypothesized that PA distensibility could provide non-invasive data useful in the follow-up of distal CTEPH. We present data showing a significant relationship between RAC and functional response to long-term sildenafil treatment in distal CTEPH. This adds further evidence showing that measurement of PA distensibility is a potentially useful marker of disease progression.

## Methods

### Patients

The local ethics review committee approved this study. Full written, informed consent was obtained from all subjects. Subjects were diagnosed with CTEPH according to standard classification.^17,18^ The methods and patients were described in detail previously.^3^ The diagnosis was made by a multidisciplinary team of PEA surgeons, physicians and radiologists using at least 2 of the following 4 imaging modalities; ventilation/perfusion scanning; conventional pulmonary angiography; computed tomography (CT) pulmonary angiography; and magnetic resonance imaging (MRI) pulmonary angiography. Subjects were enrolled with either a de novo diagnosis of distal inoperable disease or post-PEA residual distal disease and significant residual pulmonary hypertension, and were required to be clinically stable on unchanged therapy, including stable anti-coagulation for a period of 3 months. Subjects were excluded if there was any co-existing significant disease, in particular pulmonary or cardiac disease, including obstructive airway disease, pulmonary fibrosis, congenital heart disease and valvular heart disease. Patients were also excluded of they had any pathology that could interfere with the undertaking and interpreting of a 6-minute walk test (6MWT).

**Figure 1:**
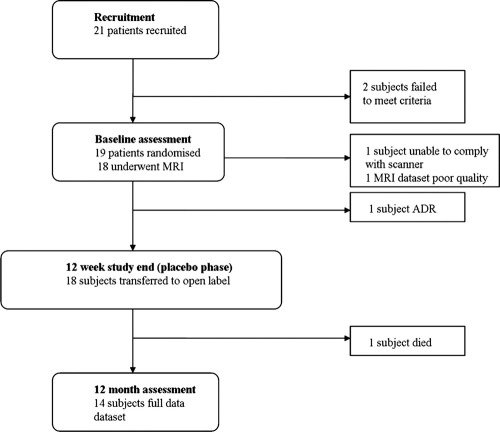
Time-line of trial protocol. ADR, adverse drug reaction.

Eighteen subjects entered into a randomized, controlled trial of sildenafil for the treatment of distal CTEPH underwent MRI scan at baseline entry into the trial. Subjects were then randomized to sildenafil titrated up to 40 mg 3 times daily or placebo for 3 months. At the end of the placebo-controlled phase, the subjects were then enrolled in an open-label phase for a further 9 months (Figure 1). The primary trial end-point was the 6MWT and secondary end-points were hemodynamics on right heart catheter, N-terminal pro–brain natriuretic peptide (NT pro-BNP), quality-of-life scores (Cambridge Pulmonary Hypertension Review, CAMPHOR) and New York Heart Association (NYHA) class. These measurements were repeated at trial end (12 weeks) and at 9 months of open-label study (1 year from trial inception). One patient was withdrawn after an adverse drug reaction, 1 patient died, 1 patient could not tolerate the scanner, and 1 patient had insufficient quality scans for analysis. The results of this trial have been reported elsewhere.^3^ We additionally report longer-term 3-year results for the therapeutic trial, although this data set is not complete.

### MRI technique

MRI was performed with a 1.5T Sigma CVi system (GE Healthcare, Milwaukee, WI). Images were acquired using a phased-array torso coil with respiratory gating and prospective electrocardiographic (ECG) gating. Subjects were ECG monitored with a T-wave–suppressing module. Multislice gradient–echo sequences were used to obtain 2-chamber, 4-chamber and axial volume stacks. All cine sequences with fast imaging employing steady-state acquisition (FIESTA) were acquired with 25 cardiac phases at 10-mm-thick slices with 128 × 256 matrices. Interleaved, velocity-encoded, phase-difference sequences were used to measure flow and area in the right pulmonary artery [RAC (%) = ((systolic area − diastolic area)/diastolic area) × 100]. MRIs were stored on a CCI picture archive and communication system (PACS) system for subsequent recall and analysis. To determine the interobserver variability, images were analyzed by 2 study-blind investigators. Interobserver variability was <10%.

### Statistics

To derive significant correlations given the small sample size and lack of Gaussian distribution Spearman's rank correlation was used. All data were analyzed using Pearson's correlations and significant correlations only designated when both methods concurred. The small sample size precluded multivariate analysis. The Mann–Whitney rank-sum test was used for between-group analysis.

## Results

**Figure 2:**
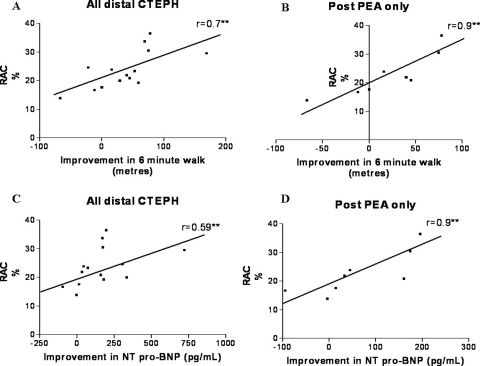
Correlations of baseline PA distensibility, as measured by relative area change (RAC) of right main pulmonary artery, with 6MWT improvement and NT pro-BNP at 1 year. PAD correlated with improvement in 6MWT for (A) all subjects and (B) post-PEA only. PAD correlated with change in NT pro-BNP for (C) all subjects and (D) post-PEA only. **p < 0.05.

Data regarding baseline demographics, hemodynamics, 6MWT and NT pro-BNP are detailed in Table 1. There was a significant correlation between pre-treatment baseline RAC and functional response at 1 year, as determined by 6MWT (*r* = 0.70, *p* = 0.006) (Figure 2). RAC at baseline also showed a significant correlation for both absolute quantitative change and percentage change in NT pro-BNP at 1 year (*r* = 0.59, *p* = 0.03 and *r* = 0.53, *p* = 0.05, respectively) (Figure 2). This relationship was particularly strong in the post-PEA residual distal disease group. RAC was highly correlated with improvement in 6MWT at 1 year (*r* = 0.91, *p* = 0.002) (Figure 2). In the residual distal post-operative disease group RAC also correlated highly with absolute and percentage change from baseline in NT pro-BNP at 1 year (*r* = 0.90, *p* = 0.005 and *r* = 0.81, *p* = 0.02, respectively) (Figure 2). There was no correlation between baseline RAC and change in mean PA pressure (*r* = 0.24, *p* = 0.57), cardiac output (*r* = −0.04, *p* = 0.9) or change in pulmonary vascular resistance (PVR; *r* = −0.14, *p* = 0.74). No other baseline variable correlated with change in 6MWT (Table 2) and this was also the case with change in NT pro-BNP. Only 5 of the subjects were on active drug during the randomized phase. The correlation between change in 6MWT and baseline RAC at 3 months in this group did not reach statistical significance (*r* = 0.53, *p* = 0.36). The placebo group showed no significant correlation at 3 months (*r* = 0.22, *p* = 0.6).

**Figure 3:**
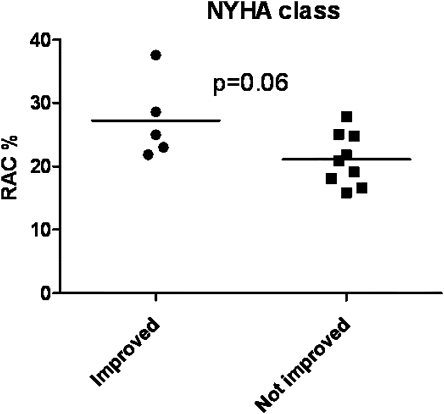
Comparison of RAC in patients who improved NHYA class at 1 year vs those who did did not improve NYHA class. RAC, relative area change (%).

The CAMPHOR quality-of-life score correlated with RAC at 3 months (*r* = 0.78, *p* = 0.001). The comparison of patients at 1 year who improved by a functional NYHA class vs those who did not or deteriorated demonstrated a higher mean RAC, although this just failed to meet statistical significance (*p* = 0.06) (Figure 3). Of interest, limited data were available at 3 years. One of the 17 patients in the trial died. This patient had an RAC of 19.1%. Of the 11 patients with CAMPHOR scores available at 3 years there was a continued improvement (mean −4.1, SD 8.4). This was not just a mean change but a CAMPHOR score improvement in 8 of the 11 subjects. There was therefore some additional data to suggest that the improvements in the trial were subjectively perceived as being of importance to the patients and that these improvements continued out to 3 years. There were limited 6MWT data available at 3 years for 7 patients. The correlation between the RAC and 6MWT for the remaining subjects (*r* = 0.42) did not reach statistical significance (*p* = 0.41), but the correlation to NT pro-BNP remained significant despite the low numbers (*r* = 0.94, *p* = 0.016).

Taking into account the small numbers involved, using a cut-off of PA distensibility of >20% across the whole study population, there was an 87.5% sensitivity (95% CI: 45% to 100%) and a 66.7% specificity (95% CI: 22% to 96%) for improvement in 6MWT of >40 meters. In the post-operative distal CTEPH group, again with a cut-off of 20%, this gave a sensitivity of 100% (95% CI: 40% to 100%) and specificity of 100% (95% CI: 40% to 100%) for improvement in 6MWT over 40 meters. There was no significant difference in hemodynamic or functional profile between those with a distensibility preserved at >20%, and those at <20% (Table 3).

## Discussion

To our knowledge, this is the first trial to look at a distinct population of patients with predominantly distal CTEPH and the potential importance of the proximal vasculature in response to medical treatment. Very recent work has demonstrated a clear correlation between exercise capacity and MRI measurement of distensibility.^19,20^ We have now presented data that suggest this may extend to the response to treatment with a significant correlation between PA distensibility, as measured by RAC at baseline, and functional improvement, as measured by 6MWT, after 1 year of an RCT and open-label phase of sildenafil treatment. It is encouraging that the 3-year data from this cohort of patients continue to demonstrate that these patients seem to have done remarkably well on targeted treatment despite their poor historic prognosis. This is in line with data we have recently presented from the whole UK population.^21^

The RAC was the only independent baseline variable demonstrating any relation to the improvement in 6MWT. In addition, the baseline RAC correlated with the change in NT pro-BNP at 1 and 3 years. As NT pro-BNP has been shown to reflect right ventricular function,^22,23^ this relationship is therefore potentially explained by the effect of preserved distensibility on the function of the right ventricle. Baseline RAC did not correlate with change in PVR or cardiac output measured at rest; however, the most relevant cardiac output to relate to the 6MWT is on exertion, as this is a direct contributor to exercise capacity. We did not perform these measurements.

The current catheter measurements of pulmonary vascular resistance do not adequately assess right ventricular after-load and give no direct information on arterial elastance or compliance.^24^ Part of the reason these parameters have not historically been routinely measured is a lack of simple methodologic approaches.^25^ More recently, compliance has been suggested to have a direct inverse relationship to PVR,^26^ and PVR coupled with compliance correlated better with cardiac index than PVR alone. Capacitance and distensibility have been demonstrated to correlate with mortality in pulmonary hypertension.^16,27^ A very recent study looking at a mixed group of patients with pulmonary hypertension confirmed that the correlation between PA elasticity and exercise capacity was superior to traditional hemodynamic measurements, but there was no investigation of treatment response.^19^ Therefore, parameters measuring compliance and after-load have potential roles in the study and understanding of disease evolution.

In our study there are 2 potential reasons why RAC correlates with response to treatment. The first explanation is that the proximal vasculature is itself a target for the remodeling properties of sildenafil. PA distensibility would therefore reflect potential proximal vasodilation. However, the RAC measurements did not correlate with our original vasodilator studies (data not shown), although the activity of the whole circuit is perhaps not expected to correlate with a selective proximal measurement. An alternative explanation is that proximal distensibility may reflect downstream remodeling. The anti-proliferative, anti-remodeling and distal vasodilatory effects of sildenafil could therefore be most pronounced in subjects with preserved distensibility and, consequently, better preserved compliance.

There are notable weaknesses in our study. Our study population was small. This is particularly problematic for the post-PEA and distal de novo inoperable CTEPH sub-groups, which were also not well matched for either functional level as measured by quality-of-life scores, 6MWT, NT pro-BNP and hemodynamic variables. Across all of these parameters, the inoperable group had more severe disease (Table 1). However, both groups had a comparable mean RAC (Table 1), and therefore it is not simply that the post-operative group had improved hemodynamics and, as a result, preserved distensibility. It is likely that the removal of proximal organized thrombus formation resulted in altered vessel structure when compared with distal de novo disease. It is a flaw in our study that the numbers were too small for accurate statistical analysis between these 2 groups; therefore, we avoided this comparison, despite the post-PEA, in particular, showing a strong correlation with response to therapy. In addition, there is the possibility that in the post-operative group there was significantly more proximal disease left post-operatively than our imaging studies revealed. The subjects were very carefully diagnosed in a multidisciplinary meeting with up to 4 angiographic imaging modalities and all of our post-PEA patients were considered to have good technical clearance of accessible proximal disease, both at operation and on subsequent imaging. Although we believe that the categorization is accurate this remains a potential confounder. Finally, another potential criticism is that the RAC did not correlate with our original vasodilator studies or to pulmonary vascular resistance. This may be explained by the proximal nature of the measurement, which will thus give little information on the distal vascular resistance or vasoreactivity.

As a small pilot study the high predictive value of PA distensibility for improvement in 6MWT and NT pro-BNP has to be tempered by the large confidence intervals that are a result of the small numbers involved. Validating this will require an adequately powered larger study. Regardless, the demonstration of a non-invasive parameter closely correlating with therapeutic treatment response, as measured by improvement in 6MWT, and right heart function, as measured by NT pro-BNP, is of clear interest in the future management of CTEPH.

## Disclosure statement

Supported by the British Heart Foundation (to M.R.T.) and by the Cambridge NIHR Biomedical Research Centre. The study protocol was registered with the UK National Research Register database (Publication ID N0542136603).

M.R.T. received travel grants from GSK and Encysive. J.P.Z. received honoraria from GSK, Pfizer, Actelion, Encysive, United Therapeutics and Schering for speaking at educational and advisory board meetings, and holds joint grants from Actelion, Pfizer, Schering, Novartis and United Therapeutics. N.W.M. received honoraria for educational talks from Pfizer and Actelion and also holds grants from Novartis Plc. K.K.S. received educational grants from GSK, Pfizer, Actelion, Encysive and United Therapeutics. The trial drug and placebo were provided by Pfizer.

## Figures and Tables

**Table 1:** Baseline Parameters of CTEPH Subjects

	All subjects: baseline parameters (SD) (*n* = 14)	Post-PEA distal CTEPH (SD) (*n* = 8)	Inoperable distal CTEPH (SD) (*n* = 6)	Post-PEA vs inoperable (*p*-value)
Age	55.3 (15.0)	55.6 (19.1)	54.8 (8.5)	0.69
Gender (M/F)	3/11	1/7	2/4	NA
Mean PA pressure (mm Hg)	44.3 (9.6)	39.1 (6.9)	51.5 (8.2)	0.012^a^
Pulmonary vascular resistance (dynes/s/cm^5^)	720 (380)	539 (272)	961 (386)	0.036^a^
PCWP (mm Hg)	10 (3.6)	10.1 (2.9)	10.0 (4.6)	0.86
Cardiac output (liters/min/m^2^)	4.2 (1.0)	4.6 (0.85)	3.8 (1.2)	0.15
NT pro-BNP (pg/ml)	1,057 (1,262)	389 (397)	1,903 (1,550)	0.02^a^
6-minute walk test (m)	336.4 (60.3)	338.6 (79.8)	335 (51.8)	0.86
RAC	32.3 (5.6)	22.8 (7.3)	24 (2.5)	0.34

CTEPH, chronic thromboembolic pulmonary hypertension; PA, pulmonary artery; PCWP, pulmonary capillary wedge pressure; PEA, pulmonary endartectomy; NA, not applicable; NT pro-BNP, N-terminal pro–brain natriuretic peptide; RAC, relative area change.

a Statistically significant (*p* < 0.05).

**Table 2:** Baseline Variables Correlated With Improvement in 6MWT After 1 Year

	Baseline parameters (SD)	Correlation with 6MWT	*p*-value
Age	55.3 (15.0)	−0.12	0.68
Gender M/F	3/11	0.17	0.51
SpO_2_ on air (%)	94.6 (2.6)	−0.29	0.31
Systemic mean blood pressure (mm Hg)	98 (18.2)	−0.18	0.53
Mean PA pressure (mm Hg)	44.3 (9.6)	0.16	0.52
Pulmonary vascular resistance (dynes/s/cm^5^)	720 (380)	0.38	0.18
PCWP (mm Hg)	10 (3.6)	—	0.15
Cardiac output (liters/min/m^2^)	4.2 (1.0)	−0.29	0.32
NT pro-BNP (pg/ml)	1,057 (1262)	0.43	0.12
RAC	23.3 (5.6)	0.7	0.006^a^

PA, pulmonary artery; PCWP, pulmonary capillary wedge pressure; NT pro-BNP, N-terminal pro–brain natriuretic peptide; RAC, relative area change; SpO_2_, oxygen saturation; 6MWT, 6-minute walk test.

a Statistically significant (*p* < 0.05).

**Table 3:** Subjects Divided Using 20% RAC Cut-off

	Subjects with RAC >20% (*n* = 10)	Subjects with RAC <20% (*n* = 4)	*p*-value
Age	53.5 (15.0)	58.2 (8.5)	0.74
Gender (M/F)	3/7	0/4	
Mean PA pressure (mm Hg)	46.4 (3.0)	39.5 (4.8)	0.24
Cardiac output (liters/min)	4.27 (0.3)	4.4 (0.47)	0.72
Pulmonary vascular resistance (dynes/s/cm^5^)	674.2 (107.4)	451.0 (137.5)	0.27
6-minute walk test (m)	339.5 (15)	331.0 (55.8)	0.84

PA, pulmonary artery; RAC, relative area change.
